# Clinical Impact and Cellular Mechanisms of Iron Overload-Associated Bone Loss

**DOI:** 10.3389/fphar.2017.00077

**Published:** 2017-02-21

**Authors:** Viktória Jeney

**Affiliations:** Department of Medicine, University of DebrecenDebrecen, Hungary

**Keywords:** osteoporosis, RUNX2, osteoblast differentiation, Osteoclasts, iron overload disease

## Abstract

Diseases/conditions with diverse etiology, such as hemoglobinopathies, hereditary hemochromatosis and menopause, could lead to chronic iron accumulation. This condition is frequently associated with a bone phenotype; characterized by low bone mass, osteoporosis/osteopenia, altered microarchitecture and biomechanics, and increased incidence of fractures. Osteoporotic bone phenotype constitutes a major complication in patients with iron overload. The purpose of this review is to summarize what we have learnt about iron overload-associated bone loss from clinical studies and animal models. Bone is a metabolically active tissue that undergoes continuous remodeling with the involvement of osteoclasts that resorb mineralized bone, and osteoblasts that form new bone. Growing evidence suggests that both increased bone resorption and decreased bone formation are involved in the pathological bone-loss in iron overload conditions. We will discuss the cellular and molecular mechanisms that are involved in this detrimental process. Fuller understanding of this complex mechanism may lead to the development of improved therapeutics meant to interrupt the pathologic effects of excess iron on bone.

## Introduction

Nowadays, osteoporosis is a major public health problem, affecting millions of people worldwide, and it is expected to further escalate due to the progressive aging of populations. Based on demographic changes around the world and the incidence rates for hip fractures, it was estimated that the number of hip fractures occurring in the world each year will exceed 6 million by 2050 (Cooper et al., 1992).

The first multivariate analysis showing that iron overload is an independent risk factor for osteoporosis in patients with genetic hemochromatosis was published by Sinigaglia et al. (1997). This observation was confirmed by further clinical studies revealing that bone weakening—characterized by low bone mass, osteopenia, osteoporosis, altered microarchitecture and biomechanics, as well as bone fractures—is one of the common features of iron overload conditions. Moreover, a number of experimental models of iron overload have been established to confirm the deleterious effect of iron on bone metabolism.

In adults the entire skeleton is replaced in about every 10 years in a process called bone remodeling. Two major cell types are involved in bone remodeling: osteoclasts that resorb bone tissue and osteoblasts that actively synthetize new bone to fill the resulting lacunae. Osteoclasts differentiate from the monocyte/macrophage hematopoietic lineage, and osteoblasts differentiate from multipotent mesenchymal stem cells. Differentiation and activity of osteoclasts and osteoblasts must be tightly regulated in order to maintain skeletal health throughout life. There is growing evidence that excess iron disturb the delicate balance in bone remodeling.

## Detrimental role of excess iron on bone density, microarchitecture, and biomechanics

Diseases/conditions with diverse etiology, such as hemoglobinopathies, hereditary hemochromatosis and menopause, could lead to chronic iron accumulation. Case reports and clinical studies revealed that bone weakening—characterized by low bone mass, osteopenia, osteoporosis, altered microarchitecture and biomechanics, as well as bone fractures—is one of the common features of these iron overload conditions. Moreover, osteoporotic bone phenotype has been observed in diverse animal models of iron overload, suggesting a detrimental role of excess iron on bone.

### Clinical findings

#### Thalassemia bone disease

Thalassemias are inherited blood disorders due to diverse mutations in the genes encoding hemoglobin (Hb) chains (α and β) causing various degrees of anemia (Weatherall, 1997). Severity of thalassemia varies widely from mild anemia (thalassemia intermedia) through severe forms (thalassemia major) to intrauterine death. Thalassemia major patients are on regular blood transfusion therapy in order to maintain sufficient Hb levels (Weatherall, 1997). The human body lacks active mechanism to excrete excess iron, therefore repeated blood transfusions lead to iron overload in these patients. Excess iron can deposit in body organs in particularly in pancreas, liver, pituitary, and the heart. To avoid iron overload, thalassemia patients are treated with iron chelators concomitantly with the blood transfusions (Bayanzay and Alzoebie, 2016). In spite of iron chelation therapy, iron overload is a major manifestation of the disease that contributes largely to the multiple end-organ complications associated with thalassemia major.

Thalassemia-associated osteoporosis and subsequent bone fractures remained to be one of the most frequent co-morbidity in thalassemia patients (reviewed in Dede et al., 2016). According to the findings of clinical studies performed in the last 40 years, fracture rates decreased in thalassemia patients, that could be due to the improvement of iron chelation therapy (Dines et al., 1976; Exarchou et al., 1984; Finsterbush et al., 1985; Ruggiero and De Sanctis, 1998; Fung et al., 2008). A recent cohort published in 2006 reported increased prevalence of lifetime fracture rate in both thalassemia major and thalassemia intermedia patients, that could be associated with increased life expectancy of the patients and older age of the population group (Vogiatzi et al., 2006). Importantly, fracture risk correlates with severity of anemia and the frequency of blood transfusion in patients with thalassemia.

Bone mineral density (BMD) is a good predictor of fractures in thalassemia patients similarly to that of healthy subjects. Prevalence of low BMD is increasing sharply by age in thalassemia patients. For example decreased BMD is present in less than 10% of children with thalassemia (6–10 years) but more than 60% of the adult patients (>20 years) have low BMD, regardless of optimal treatment (Vogiatzi et al., 2006). Because low BMD alone cannot explain the high fracture risk described in patients with thalassemia, further studies were performed to study bone quality. Trabecular bone score, as a measure of bone quality, was found to be lower in adults with thalassemia compared to healthy individuals (Baldini et al., 2014). Similarly to that of BMD, trabecular bone score also correlated with age, suggesting that thalassemia negatively influence both bone quality and quantity (Baldini et al., 2014).

Bone is a dynamic tissue, therefore diverse mechanisms could contribute to low bone quantity and quality in thalassemia patients. Several studies suggested the involvement of increased bone resorption in the pathogenesis of thalassemia-associated bone disease (Voskaridou et al., 2001; Morabito et al., 2004; Vogiatzi et al., 2006; Angelopoulos et al., 2007). Other studies proposed that bone formation is severely impaired in patients with thalassemia. Bone histomorphometry revealed that osteoid thickness and osteoid maturation time are increased in iliac crest of children with β-thalassemia indicating impaired bone matrix maturation and defective mineralization (Mahachoklertwattana et al., 2003). Interestingly, iron deposits were detected along mineralization fronts and osteoid surfaces, and were co-localized with thickened osteoid seams (Bordat et al., 1993; Mahachoklertwattana et al., 2003).

#### Sickle cell bone disease

Sickle cell disease (SCD) is an inherited autosomal recessive disorder, caused by a single nucleotide mutation in the Hb β-chain coding gene (*HBB*). SCD is one of the most prevalent genetic blood disorders worldwide affecting more than 300,000 annual births. SCD is associated with significant morbidity and shortened life span (45–65 years; Hamideh and Alvarez, 2013).

Bone involvement is very common in SCD and can manifest in diverse ways including but not limited to bone and joint pain, vertebral bone deformities, osteopenia and osteoporosis and pathological fractures (reviewed in Almeida and Roberts, 2005; Osunkwo, 2013). Low BMD in present in more than 70% of SCD patients at the age of 30 years that predispose those patients to high risk of fractures and vertebral collapse (Miller et al., 2006; Sadat-Ali and Al Elq, 2007; Sarrai et al., 2007; Sadat-Ali et al., 2008). Although the association between SCD and low BMD is well established, there are very few studies addressing the reason of low BMD in SCD. Recently, Baldanzi et al. (2011) analyzed 65 SCD patients and found that low BMD was associated with increased hemolysis, which was characterized by increased lactate dehydrogenase levels and reticulocyte counts as well as decreased hemoglobin levels.

Iron status of SCD patients is quite controversial, as some reports suggest that SCD is associated with iron overload while others describe SCD as a condition of iron deficiency (Koduri, 2003; Koren et al., 2010). This might be due to the abnormal compartmentalization of iron in SCD patients that can lead to hepatic, splenic, and renal siderosis but no iron stores in the bone marrow, simultaneously (Natta et al., 1985). Moreover, autopsy studies revealed huge individual variations in iron deposits in SCD patients (Natta et al., 1985). Nevertheless, a recent cross-sectional study revealed that about 70% of SCD patients with high serum iron had lower bone mass, thus suggesting a negative effect of excess iron—if present in SCD—on bone physiology (Sadat-Ali et al., 2011).

#### Bone abnormalities in hereditary hemochromatosis

Hemochromatosis refers to a group of inherited disorders characterized by increased dietary iron uptake, which in some cases can lead to severe tissue iron overload. Up to date five types of hereditary hemochromatosis are described, each caused by mutations in different genes involved in iron metabolism. The most common form of hereditary hemochromatosis (HH) in Caucasian population is due to the Cys282Tyr mutation of the *HFE* gene (Feder et al., 1996; McLaren and Gordeuk, 2009). Other types of hereditary hemochromatosis are caused by mutations in the transferrin receptor-2 gene (*TfR2*) (Camaschella et al., 2000), hemojuvelin gene (*HJV*) (Papanikolaou et al., 2004), hepcidin gene (*HAMP*) (Roetto et al., 2003), and the ferroportin gene (*SCL40A1*) (Pietrangelo, 2004).

In the last 50 years evidence piled up suggesting the adverse effect of HH on joints and bones (reviewed in Guggenbuhl et al., 2011a). Case reports and small-scale studies suggested that HH is associated with osteoporosis, and that the development of osteoporosis correlates with the severity of iron overload independently of cirrhosis and hypogonadism (Diamond et al., 1989; Sinigaglia et al., 1997; Guggenbuhl et al., 2005; Angelopoulos et al., 2006). Recently, the incidences of osteoporosis and osteopenia were determined in patients with HH. These studies revealed that about 25–34% of HH patients have osteoporosis and 40–80% of the patients have osteopenia (Guggenbuhl et al., 2005; Valenti et al., 2009). A recent case-control study with the involvement of ~300 HH patients and ~300 age-, and sex-matched controls clearly showed that HH is associated with increased prevalence of osteoporosis (Richette et al., 2010). Osteoporotic fractures were described in some HH patients (Eyres et al., 1992; Duquenne et al., 1996), and the above-mentioned study showed that patients with HH showed an increased prevalence, although not significant, of wrist and vertebral fractures. Moreover the same study revealed that the severity of iron overload was associated with wrist and vertebral fractures.

#### Postmenopausal osteoporosis

Postmenopausal osteoporosis is a systemic bone metabolism disorder affecting 30% of women over the age of 50 (reviewed recently in Black and Rosen, 2016). The disease is characterized by progressive bone loss and subsequent increase in the risk of fractures. Among postmenopausal white women the lifetime risk of hip fracture is 15–20%, and the risk of any osteoporotic fractures is about 50% (Cummings et al., 1989; Cummings and Melton, 2002).

Estrogen deficiency, a prominent feature of menopause, has been considered as the main cause of menopausal symptoms and disorders. For example, estrogen deficiency has been shown to change bone metabolism via promoting bone resorption and decreasing bone formation, and therefore contribute to postmenopausal osteoporosis (Bowring and Francis, 2011). Menopausal transition is a complex process, in which besides hormonal shifts, iron metabolism is also altered. Parallel with the decline in estrogen level, a two- to three-fold increase in serum ferritin concentration was detected in postmenopausal women (Milman and Kirchhoff, 1992; Zacharski et al., 2000). Although elevated iron as a result of menopause is in the physiologic range, mounting evidence suggest that besides estrogen deficiency, iron/ferritin accumulation affect the health of postmenopausal women (Jian et al., 2009). Recent studies investigated the association between plasma ferritin level and BMD. Kim et al. (2012) performed a 3-year retrospective longitudinal study with the involvement of 940 postmenopausal women in which they determined annual bone loss rates and plasma ferritin levels. They found that rates of bone loss were significantly accelerated in a dose-dependent manner across increasing ferritin quartile categories. Results of a population-based, cross-sectional study from the Korea National Health and Nutrition Examination Surveys provided further evidence that there is an inverse correlation between serum ferritin level and BMD in women ≥45 years of age (Kim et al., 2013). These studies provided clinical evidence that increased total body iron stores could be an independent risk factor for accelerated bone loss in postmenopausal women. Interestingly, bisphosphonates, a widely used agent to treat osteoporosis in postmenopausal women has been shown to decrease plasma ferritin levels significantly (Feldbrin et al., 2016). Whether this effect of bisphosphonates, contributes to its anti-osteoporosis action, needs to be investigated in the future.

### Animal models

As it was discussed in the previous chapter, iron overload associates with osteoporosis and bone fractures, but it remained elucidated whether this is due to a direct effect of iron on bone metabolism, or is the result of simultaneous endocrine alterations, or the effect of chronic illness itself. To answer this question Tsay et al. (2010) developed a mouse model of iron overload in which C57/BL6 mice were injected with iron dextran for 2 months. This approach resulted in tissue iron overload in various organs, including liver, spleen, and heart which was associated with osteoporosis (Tsay et al., 2010). Iron overload-driven bone loss was associated with increased production of reactive oxygen species (ROS) and inflammatory cytokines such as tumor necrosis factor-α (TNF-α) and interleukin-6 (IL-6) (Tsay et al., 2010). The free radical scavenger N-acetyl-L-cysteine (NAC) could partially prevent the development of iron overload-triggered bone abnormalities, highlighting the critical role of ROS in the mechanism of bone loss (Tsay et al., 2010).

Bone phenotype was studied in several genetic iron overload mouse models as well. For example both heterozygous and homozygous th3 thalassemia mice as well as hemizygous β-globin knockout and β^IVSII−654^ knockin thalassemic mice exhibited decreased trabecular bone fraction and thickness, lower number of trabeculae, thinner cortices and increased marrow area (Vogiatzi et al., 2010; Thongchote et al., 2014, 2015). These changes in bone microarchitecture were associated with decreased bone turnover and poor biomechanical properties in the thalassemia mice models (Vogiatzi et al., 2010; Thongchote et al., 2014). Regarding sickle cell disease, deleterious structural changes in the bone microarchitecture and mechanics as well as decreased BMD were detected in aged transgenic sickle cell disease and sickle cell trait mice (Green et al., 2015; Xiao et al., 2016).

Studies accomplished using genetic mice models of hemochromatosis also supported the negative influence of excess iron on bone metabolism. For example Hfe knockout (Hfe^−/−^) mice had a phenotype of osteoporosis with low bone mass and alteration of the bone microarchitecture (Guggenbuhl et al., 2011b; Doyard et al., 2016). Similar bone phenotype was described in hepcidin deficient (Hamp1^−/−^) mice (Shen et al., 2014; Sun et al., 2014) and in zebrafish (Jiang et al., 2016). The mechanism and the pathophysiological role of iron accumulation in postmenopausal osteoporosis were also investigated using animal models. In a recent study Xiao et al. (2015) found that iron overload aggravated ovariectomy-induced bone-loss in mice. Studies revealed that iron-induced oxidative stress could contribute to deterioration of bone metabolism and subsequent osteoporosis after menopause (Isomura et al., 2004; Xiao et al., 2015).

Bone remodeling, the cycle of bone resorption and bone formation, is critically important to maintain skeletal health throughout life. In bone remodeling osteoclasts resorb bone tissue and osteoblasts actively synthetize new bone tissue. Growing evidence suggest that excess iron disturb the delicate balance between bone resorption and bone formation which can manifest as low bone mass, osteopenia, osteoporosis, or bone fractures. Studies revealed that excess iron can influence both osteoclast and osteoblast functions.

## Cellular mechanisms

**Figure 1:**
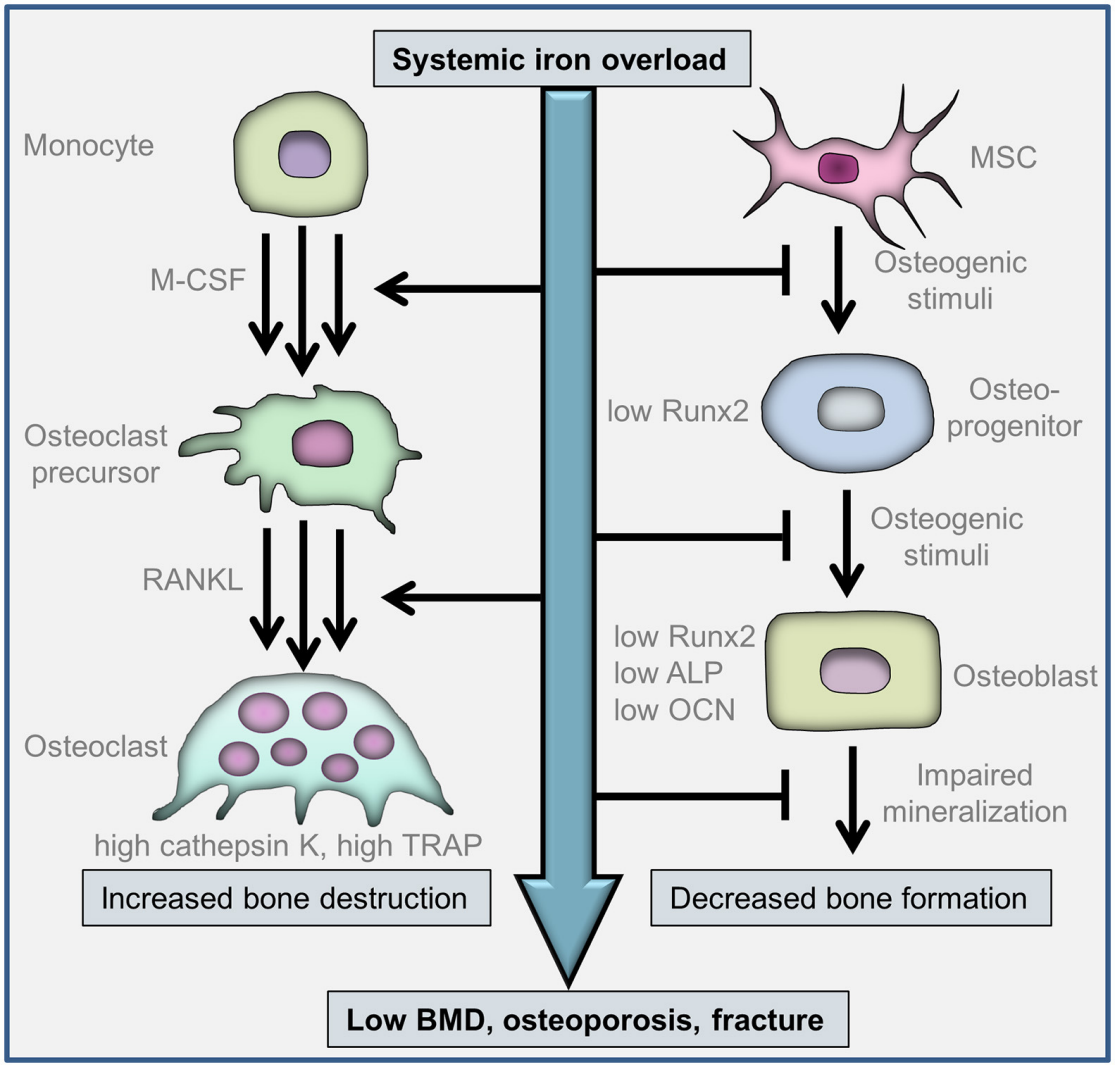
**Cellular mechanisms involved in iron overload-associated bone-loss**. Osteoclasts are differentiated from myeloid cells of the monocyte/macrophage lineage. The differentiation is initiated by macrophage colony-stimulating factor (M-CSF) and receptor activator of nuclear factor κB ligand (RANKL). Multinuclear osteoclasts are formed via the fusion of mononuclear osteoclast precursors. Iron facilitates osteoclast differentiation, activation and bone resorption. Osteoblasts derive from multipotent mesenchymal stem cells (MSCs). Iron inhibits osteogenic differentiation of MSCs via the downregulation of the master osteogenic transcription factor runt-related transcription factor 2 (Runx2). Iron impairs Runx2-dependent upregulation of osteoblast-specific proteins, *e.g.*, alkaline phosphatase (ALP) and osteocalcin (OCN), and inhibits extracellular matrix mineralization. Increased bone destruction by osteoclasts and decreased bone formation by osteoblasts contribute to bone loss in patients with systemic iron-overload.

Bone is a metabolically active tissue that undergoes continuous remodeling with the involvement of osteoclasts that resorb mineralized bone, and osteoblasts that form new bone matrix (Zaidi, 2007). *In vivo* observations revealed that both increased bone resorption and decreased bone formation are involved in the pathological bone-loss in iron overload conditions (Figure 1). Osteoclasts derive from the monocyte/macrophage hematopoietic lineage, and osteoblasts differentiate from multipotent mesenchymal stem cells. The *in vitro* effect of excess iron on osteoclast and osteoblast functions and their differentiation processes have been studied and will be discussed in this chapter.

### The effects of iron overload in bone resorption

#### Influence of iron on osteoclast differentiation and activity: *In vitro* findings

Osteoclasts are differentiated from myeloid cells of the monocyte/macrophage lineage. The differentiation is initiated by osteoclastogenic cytokines, i.e., macrophage colony-stimulating factor (M-CSF) and receptor activator of nuclear factor κB ligand (RANKL) (Teitelbaum and Ross, 2003). RANKL is controlled by osteoprotegerin (OPG), a decoy receptor for RANKL, which inhibits osteoclastogenesis. Therefore, the RANKL/OPG pathway is the dominant regulator of osteoclast proliferation and activation (Hofbauer et al., 2004), and the RANKL/OPG ratio is an important determinant of bone mass and skeletal integrity (Boyce and Xing, 2007). Following the formation of mononuclear pre-osteoclasts, they fuse and form the multinuclear mature osteoclasts which die of apoptosis within a few days. Osteoclasts are effector cells that are involved in breaking down bone tissue. They seal the resorption bays, and form a specialized cell membrane, the ruffled border at the site of active bone resorption. Osteoclast release hydrogen ions into the resorption cavity, to acidify the milieu, that facilitates the dissolution of the inorganic bone matrix (Teitelbaum and Ross, 2003; Ikeda and Takeshita, 2016). Additionally, activated osteoclasts secrete proteolytic enzymes, such as cathepsins and matrix metalloproteinases, which degrade bone matrix proteins (Teitelbaum and Ross, 2003; Ikeda and Takeshita, 2016). Mature osteoclasts highly express tartrate-resistant acid phosphatase (TRAP) that they release upon active bone resorption. Consequently, TRAP 5b is a useful serum marker of bone resorption (Halleen et al., 2000).

Accumulating evidence suggest that iron facilitate osteoclast differentiation, activation and bone resorption (Figure 1). Osteoclasts have high energy demand which is fueled by a lot of mitochondria (Roodman, 2009). Mitochondrial biogenesis is an iron-dependent process, because the proteins of the respiratory chain are iron-containing proteins. Osteoclast differentiation was found to be associated with the induction of transferrin receptor 1 (Tfr1) and increased iron uptake (Ishii et al., 2009; Roodman, 2009). Tfr1-mediated iron uptake facilitated osteoclast differentiation and bone-resorbing activity, whereas iron chelation inhibited osteoclastic bone resorption (Ishii et al., 2009). This finding was confirmed by Jia et al. (2012) who showed that ferric ion promoted RANKL-induced osteoclast formation in both RAW264.7 cells and bone marrow-derived macrophages. Iron catalyzes the formation of ROS and this effect was found to be critical in iron-mediated promotion of osteoclast differentiation (Jia et al., 2012). Iron chelation approaches provided further evidence to establish the central role of iron in osteoclastogenesis. Cornish et al. (2004) found that lactoferrin, an iron-binding glycoprotein, at a concentration of 100 μg/ml, completely arrested osteoclastogenic differentiation of monocytes, which was associated with decreased expression of RANKL. Iron overload conditions are associated with elevated RANKL/OPG ratio (Morabito et al., 2004), whereas the iron-chelating lactoferrin has been shown to improve bone density via decreasing RANKL/OPG ratio (Hou et al., 2012).

Besides affecting osteoclastogenesis, growing evidence suggest that iron influences mature osteoclast activity and bone resorption as well. Early studies showed that inhibition of TRAP activity in osteoclasts abolishes bone resorption (Zaidi et al., 1989). TRAP contains 2 iron atoms/molecule, and its activity is rapidly inhibited by a ferric chelator but not by a ferrous chelator, suggesting that ferric iron is essential for enzymatic catalysis (Hayman et al., 1989; Hayman and Cox, 1994). Additionally, Alcantara et al. (1994) showed that TRAP mRNA contains an iron regulatory element at the 5′-flanking sequence, therefore TRAP expression is regulated by iron at the level of gene transcription. Overall, these studies revealed that iron excess can facilitate bone resorption by facilitating osteoclastogenesis and by increasing osteoclast activity. These mechanisms could contribute to unbalanced bone remodeling in iron-overload diseases.

#### Influence of iron overload on bone resorption: *In vivo* findings

Iron overload-associated bone weakening was shown by several experimental animal models. In some of these works the authors tried to dissect whether the observed bone phenotype is associated with increased bone resorption or decreased bone formation (Table 1). Based on bone histomorphometry, Tsay et al. (2010) revealed that the osteoporotic phenotype of iron overloaded C57/BL6 mice is associated with increased number of osteoclasts in the bone tissue. Moreover, the study of Guggenbuhl et al. (2011b) on Hfe^−/−^ mice led to the same conclusion that iron-overload mediated bone loss is due to elevated numbers of osteoclasts. Furthermore, Sun et al. (2014) found that the level of C-telopeptide of type-I collagen, a serum bone resorption marker, is elevated in Hamp^−/−^ mice, suggesting that increased osteoclast activity contributes to bone-loss in those mice. These studies suggested that increased bone resorption rather than decreased bone formation is the underlying mechanism of iron overload-associated bone loss (Table 1).

**Table 1:** **Studies addressing the effects of iron overload in bone remodeling**.

**Experimental model**	**Major findings**	**Experimental approach**	**References**
C57BL/6 mice iron dextran treatment	Increased osteoclast number	Bone histomorphometry	Tsay et al., 2010
Hfe^−/−^ mice	Increased osteoclast number	Bone histomorphometry	Guggenbuhl et al., 2011b
Hamp1^−/−^ mice	Increased osteoclast activity	ELISA; serum C-telopeptide of type I collagen	Sun et al., 2014
th3/th3 thalassemia mice	Decreased bone formation and decreased bone resorption	Bone histomorphometry	Vogiatzi et al., 2010
β-globin knock-out thalassemic mice	Decreased bone formation and increased bone resorption	Bone histomorphometry	Thongchote et al., 2015
Townes transgenic sickle mice	Decreased terminal differentiation of osteoblasts	qRT-PCR; ALP, OCN, Runx2, osterix mRNA	Xiao et al., 2016
Hfe^−/−^ mice	Decreased bone formation Reduced number of active osteoblasts	Bone histomorphometry, histology	Doyard et al., 2016
Hamp1^−/−^ mice	Decreased osteoblast activity	ELISA; serum OCN	Shen et al., 2014
Hamp1 knockdown zebrafish	Decreased osteoblast activity	qRT-PCR; Runx2a, Runx2b, osterix mRNA	Jiang et al., 2016
Postmenopausal Wistar rats	Decreased bone formation	ELISA; serum OCN, serum ALP activity	Isomura et al., 2004
C57BL/6 mice iron dextran treatment	Decreased osteogenic differentiation of osteoprogenitors	qRT-PCR; Runx2 mRNA	Balogh et al., 2016

*Hfe^−/−^ hemochromatosis protein deficient, Hamp^−/−^ hepcidin deficient, ELISA enzyme-linked immunosorbent assay, qRT-PCR quantitative real time PCR, ALP alkaline phosphatase, OCN osteocalcin, Runx2 Runt-related factor 2*.

### The effects of iron overload in bone formation

#### Influence of iron on osteoblast differentiation and activity: *In vitro* findings

Osteoblasts derive from multipotent mesenchymal stem cells (MSCs), which migrate to the site of injury, proliferate and differentiate. Osteogenic potential of these bone marrow-derived MSCs (BMSCs) is of crucial importance for proper remodeling and/or healing, which is highlighted by the recent discovery, that osteoporosis is associated with increased number, but low osteogenic potential of circulating MSCs (Dalle Carbonare et al., 2009). Many secreted differentiation factors, including transforming growth factor-beta 1, fibroblast growth factor, bone morphogenetic protein, wingless proteins, Indian HedgeHog can activate those signaling mechanisms that involved in osteogenic differentiation of MSCs (Hayrapetyan et al., 2015). These diverse pathways all converge on the master osteogenic transcription factor, runt-related transcription factor 2 (Runx2) (Franceschi et al., 2003). The absence of Runx2 in mice results impaired bone formation, the lack of differentiated osteoblasts, and eventually leads to death shortly after birth (Komori et al., 1997; Otto et al., 1997). Transcription of the major bone-specific proteins, such as osteocalcin (OCN), osteopontin, bone sialoprotein (BSP), α1 type I collagen (Col I) and alkaline phosphatase (ALP) are under the control of Runx2 (Ducy et al., 1997). Recently, the critical involvement of ROS in osteogenesis was established, as a common denominator of the diverse osteogenic signaling pathways (Atashi et al., 2015). Studies revealed that tightly regulated levels of ROS are critical in osteogenic differentiation of MSCs (Atashi et al., 2015).

Recently, Balogh et al. (2016) showed that iron selectively inhibits osteogenic differentiation of BMSCs without influencing adipogenic and chondrogenic differentiation. Iron at the concentration of 50 μmol/L completely abolished osteogenic stimuli-induced upregulation of Runx2 and its target genes OCN and ALP (Balogh et al., 2016). The anti-osteogenic effect of iron was mimicked by exogenously administered ferritin, the major intracellular iron storage protein (Balogh et al., 2016). Some other studies, originated from the field of stem cell-based therapeutics, also supported the inhibitory nature of iron on osteogenic differentiation of MSCs. For example it was reported that superparamagnetic iron oxide nanoparticles—that are widely used for labeling and *in vivo* tracking of MSCs—increased intracellular iron content in MSCs and impaired osteogenic differentiation of human MSCs (Chen et al., 2010; Chang et al., 2012). Moreover, the iron chelator desferrioxamine (DFO) abolished the anti-osteogenic effect of superparamagnetic iron oxide nanoparticles, demonstrating the crucial role of free iron in the inhibition of osteogenic differentiation of MSCs (Chen et al., 2010). Recently, the involvement of Wnt/β-catenin pathway—a critical regulator of bone remodeling—was examined in iron-modulated osteogenic differentiation of osteoblast progenitors. Qu et al. (2008) showed that DFO increases ALP activity and calcium deposition of MSCs which was accompanied by promoted phosphorylation of glycogen synthase kinase-3β (GSK-3β) and increased β-catenin levels. Baschant et al. (2016) showed that DFO induced the expression of Wnt5a and identified Wnt5a as a key target for the pro-osteogenic effect of DFO. Messer et al. (2009) investigated how excess iron influence differentiation and function of fetal rat calvaria cells. They showed that osteoblast phenotypic gene markers were downregulated, and the capacity of iron-loaded cells to form mineralized bone nodules was decreased. This work suggested that not only osteogenic differentiation, but activity and extracellular matrix mineralization of mature osteoblasts are also negatively influenced by excess iron.

The question whether excess iron affect osteoblast function arose more than 25 years ago, and initial studies showed that iron at high concentration diminish cellular proliferation and function of osteoblast-like osteosarcoma cells (Diamond et al., 1991). Then Messer et al. (2009) showed that excess iron (0–10 μmol/L) results in rapid and sustained down-regulation of transferrin receptor and a later up-regulation of light and heavy chain ferritins in osteoblasts obtained from fetal rat calvaria. Concurrently with these changes, osteoblast phenotype gene markers, ALP, OCN, BSP, and Col I were suppressed by day 15, and a decreased number of mineralized nodules at day 20 were observed (Messer et al., 2009). They also observed apoptotic events within 24 h of iron loading (Messer et al., 2009). Zarjou et al. (2010) showed that iron (0–50 μmol/L) dose-dependently downregulated the expression of Runx2 and its target genes OCN and ALP in mature human osteoblasts. This effect of iron was accompanied by diminished mineralization of the extracellular matrix of osteoblasts. That study also demonstrated that iron-mediated suppressions of osteoblast activity and extracellular matrix mineralization are provided by iron-induced upregulation of ferritin, and its ferroxidase activity (Zarjou et al., 2009, 2010). This observation was supported by further investigations in which the authors showed that iron/ferritin inhibits osteoblast activity and extracellular matrix mineralization induced by bone morphogenetic protein 2 or β-glycerophosphate and activated vitamin D_3_ (Yang et al., 2011; Becs et al., 2016). Studying the underlying signaling mechanism of iron-mediated inhibition of osteoblast activity revealed that excess iron decreased mRNA level of HedgeHog Interacting Protein Like-2 gene, encoding an inhibitor of the HedgeHog signaling pathway (Doyard et al., 2012).

#### Influence of iron overload on bone formation: *In vivo* findings

In the last decade several *in vivo* animal studies revealed that iron overload influence bone formation (Table 1). Bone histomorphometry showed decreased bone formation in both th3 thalassemia mice and hemizygous β-globin knockout mice (Vogiatzi et al., 2010; Thongchote et al., 2015). Doyard et al. (2016) also showed reduced number of active osteoblasts in Hfe^−/−^ mice. Xiao et al. (2016) found low mRNA expressions of osteoblast markers including ALP, Runx2, osterix, and OCN in tibia of Townes transgenic sickle mice, and concluded that iron overload reduced terminal differentiation of osteoblasts in these mice, Shen et al. reported decreased OCN serum levels in hepcidin deficient mice, whereas Jiang et al. (2016) showed decreased Runx2a, Runx2b, and osterix mRNA levels in hepcidin knockdown zebrafish. These studies suggested that osteoporotic phenotype associated with hepcidin deficiency, is due to decreased osteoblast activity and delayed mineralization. Recently, Balogh et al. (2016) showed that iron overload in C57BL/6 mice, is associated with decreased osteogenic commitment and differentiation potential of compact-bone resident osteoprogenitor cells, characterized by decreased Runx2 mRNA level.

## Effect of chelation therapy on iron overload-associated bone disease

Iron chelation therapy is the main treatment of patients with systemic iron overload (Fabio et al., 2007; Kalpatthi et al., 2010; Maggio et al., 2011). Currently three main iron chelators are used in the clinical practice, i.e., DFO, deferiprone, and deferasirox (Maggio, 2007). Effectiveness of these iron chelators were extensively studied with the use of different outcomes, including ejection fraction, liver iron content, myocardial iron concentration, serum ferritin, and urinary iron excretion (reviewed in Maggio et al., 2011). Although the pathogenesis of bone disease in iron-overload patients is multifactorial, the direct detrimental effects of excess iron in bone physiology and remodeling are well established. Therefore, iron chelation therapy might be implicated in the prevention or management of iron-overload-associated bone disease, although the number of studies that specifically addressed this question remained limited. A small study of Christoforidis et al. (2007) with the involvement of 35 thalassemia patients revealed that optimal chelation therapy prevented the manifestation of osteopenia/osteoporosis in the first two decades of life. Casale et al. (2014) investigated the effect of long term deferasirox therapy on bone disease in adult β-thalassemia major patients. In that retrospective cohort study, 86 transfusion-dependent thalassemia major patients were involved, who were treated with deferasirox once daily, for a median duration of 6.5 years (Casale et al., 2014). They found that BMD of the lumbar spine increased, and the number of patients with lumbar spine osteoporosis decreased, upon chelation therapy. Importantly, the observed changes were independent of bisphosphonate therapy, hormonal replacement therapy, and calcium or vitamin D supplementation, suggesting a direct positive effect of iron chelation on bone status in those patients (Casale et al., 2014). In a recent study, Poggi et al. (2016) compared the long-term effects of different iron chelation regimens, in preventing bone disease in patients with β-thalassemia major. In agreement with the previously mentioned study (Casale et al., 2014), they observed a significant increase in mean BMD T-score and a decrease in the prevalence of osteoporosis in the patients receiving deferasirox (Poggi et al., 2016). Surprisingly, the other iron chelation regimens (DFO and deferiprone alone, or in combination) used in this study, did not provide any beneficial effects on BMD (Poggi et al., 2016). Although these investigations suggested that proper iron chelation therapy prevents iron overload-associated bone disease, a recent study performed by the Thalassemia Clinical Research Network concluded that bone disease remained a frequent, and unresolved problem in thalassemia (Vogiatzi et al., 2009).

## Conclusions

Iron overload, can be of hereditary or acquired origin, is an excess iron in the body that can accumulate in vital organs. Iron overload increases the risk for liver disease, heart attack or heart failure, and endocrine disorders. Additionally, this condition is frequently associated with a bone phenotype; characterized by low bone mass, osteoporosis/osteopenia, altered microarchitecture and biomechanics, and increased incidence of fractures. Bone metabolism is a complex sequence of bone resorption by osteoclasts, and bone formation by osteoblasts. In order to maintain skeletal health, these processes should be very well harmonized. Excess iron disturbs the delicate balance between bone resorption and bone formation, resulting in bone weakening. Evidence suggests that both increased bone resorption, and decreased bone formation are involved in the pathological bone-loss in iron overload conditions.

Iron overload, if it remains untreated, can cause premature death. Iron chelators are in the first line of therapeutic interventions to prevent iron accumulation in vital organs. With chelation therapy, the life expectancy of thalassemia major patients is vastly improved, whereas early treatment can confer normal life expectancy for patients with hereditary hemochromatosis. As life expectancy of patients with iron-overload increases, the prevalence of bone disease also increases, suggesting that the current therapeutic interventions cannot confer full protection for the bones. Further studies needed to investigate the effects of iron chelation therapies on bone metabolism. Fuller understanding of this complex mechanism may lead to the improvement of current therapeutic interventions meant to interrupt the pathologic effects of excess iron on bone.

## Author contributions

VJ conceptualized the article, performed the literature search, draw the figure and wrote the manuscript.

### Conflict of interest statement

The author declares that the research was conducted in the absence of any commercial or financial relationships that could be construed as a potential conflict of interest.
